# Draft Genome Sequence of Plant Growth-Promoting and Drought-Tolerant Bacillus altitudinis FD48, Isolated from Rice Phylloplane

**DOI:** 10.1128/genomeA.00019-18

**Published:** 2018-03-01

**Authors:** Sowmya Kumaravel, Sugitha Thankappan, Sridar Raghupathi, Sivakumar Uthandi

**Affiliations:** a Department of Agricultural Microbiology, Tamil Nadu Agricultural University, Coimbatore, Tamil Nadu, India

## Abstract

The genome sequence of a temperature-tolerant strain, Bacillus altitudinis FD48, is described here. The reads were assembled into contigs with a total size of 3.7 Mb. The genome information will aid in understanding its role in alleviating stress in crop plants as a potential bioinoculant for agricultural applications.

## GENOME ANNOUNCEMENT

Bacillus altitudinis FD48, a stress-tolerant plant growth-promoting bacterium (PGPB), isolated from rice phylloplane, possessed a 3.7-Mb genome which harbors gene clusters for heat shock proteins (chaperonin), cysteine synthesis, trehalose synthesis, stress response proteins, and siderophore biosynthesis. B. altitudinis FD48, a Gram-positive bacterium isolated from the leaf surface of rice genotype ADT 43 by a leaf imprinting technique (1), not only survives under induced drought conditions (−0.69 MPa) but also confers various PGP benefits, like solubilization of phosphorus, production of plant growth hormones (indole-3-acetic acid [IAA], cytokinin), exopolysaccharides, 1-aminocyclopropane-1-carboxylate (ACC) deaminase activity, and saline tolerance up to 7.5%.

Genomic DNA (gDNA) was extracted from an overnight monoculture of B. altitudinis FD48 using the standard protocol of the cetyltrimethylammonium bromide (CTAB) method (2), with minor modifications. High-quality gDNA was subjected to TruSeq library preparation and was sequenced using an Illumina HiSeq 4000 sequencer. Quality control (QC) of the raw data was done to qualify reads with a Phred score of >30 for downstream analysis. Reads that passed QC were subjected to *de novo* assembly using Velvet (version 1.2.10), followed by genome finishing using the CONTIGuator tool (3, 4), resulting in a genome size of 3.7 Mb with 15 scaffold sequences and 41.19% G+C content. The *N*_50_ contig size was 976,391 bp.

A total of 4,029 predicted genes, including 3,964 protein-coding genes (CDSs) and 65 non-protein-coding genes, were observed, along with 2,882 characterized proteins and 1,080 hypothetical/putative proteins. The 16S rRNA gene sequence of the isolate FD48 showed 99% similarity to that of B. altitudinis.

The genome of B. altitudinis FD48 comprises several genes related to plant growth promotion mechanisms, such as those for the biogenesis of organic acids involved in inorganic phosphorus solubilization (glucose dehydrogenase, citrate synthase, and lactate dehydrogenase) (5). Additionally, genes responsible for flagellar motility, chemotaxis, and biofilm synthesis, which allow B. altitudinis FD48 to move toward plant exudates, thereby facilitating adhesion to plant surfaces, in addition to genes related to growth-stimulating volatile compounds and sporulation, were encountered. In addition, the annotated genome has several genes for components of iron and siderophore uptake systems, nitrogen metabolism, and various antibiotic resistance gene clusters. Interestingly, various stress regulatory and temperature tolerance genes were noted, along with those for osmolyte production (proline and glycine betaine), cold shock proteins, heat shock proteins, and resistance to heavy metals.

### Accession number(s).

The complete genome sequence of strain FD48 has been deposited in GenBank under the accession number CP025643.
